# Better soon than never: climate change induces strong phenological reassembly in the flowering of a Mediterranean shrub community

**DOI:** 10.1093/aob/mcad193

**Published:** 2023-12-14

**Authors:** Daniel Pareja-Bonilla, Montserrat Arista, Leonor Patrícia Cerdeira Morellato, Pedro Luis Ortiz

**Affiliations:** Departamento de Biología Vegetal y Ecología, Facultad de Biología, Universidad de Sevilla, Sevilla, Spain; Departamento de Biología Vegetal y Ecología, Facultad de Biología, Universidad de Sevilla, Sevilla, Spain; Center for Research on Biodiversity Dynamics and Climate Change and Department of Biodiversity, Phenology Lab, UNESP - São Paulo State University, Biosciences Institute, São Paulo, Rio Claro, Brazil; Departamento de Biología Vegetal y Ecología, Facultad de Biología, Universidad de Sevilla, Sevilla, Spain

**Keywords:** Mediterranean shrub community, flowering phenology, climate change, ecological mismatching, long-term phenology, emerging ecological interactions, temperature rise, flowering order, co-flowering patterns, phenological reassembly

## Abstract

**Background and Aims:**

Flowering is a key process in the life cycle of a plant. Climate change is shifting flowering phenologies in the Northern Hemisphere, but studies with long data series at the community level are scarce, especially those considering the consequences of phenological changes for emerging ecological interactions. In the Mediterranean region, the effects of climate change are stronger than the global average and there is an urgent need to understand how biodiversity will be affected in this area.

**Methods:**

In this study, we investigated how the entire flowering phenology of a community comprising 51 perennial species from the south of the Iberian Peninsula changed from the decade of the 1980s to the 2020s. Furthermore, we have analysed the consequences of these changes for flowering order and co-flowering patterns.

**Key Results:**

We have found that the flowering phenology of the community has advanced by ~20 days, which is coherent with the increasing temperatures related to climate change. Individual species have generally advanced their entire flowering phenology (start and end) and increased their flowering duration. The early flowering has resulted in a re-organization of the flowering order of the community and generated new co-flowering assemblages of species, with a slight trend towards an increase of shared flowering time among species.

**Conclusions:**

The advanced flowering phenology and changes in flowering duration reported here were of unprecedented magnitude, showcasing the extreme effects of climate change on Mediterranean ecosystems. Furthermore, the effects were not similar among species, which could be attributed to differences in sensitivities of environmental cues for flowering. One consequence of these changes in flowering times is ecological mismatches, indicated by changes in the flowering order and co-flowering between decades. This new scenario might lead to new competitive or facilitative interactions and to the loss or gain of pollinators.

## INTRODUCTION

Flowering phenology, or the recurrent timing of flower development and anthesis, is one of the most important steps in the life cycle of a plant, with implications for reproductive success and overall fitness. However, climate change is affecting flowering phenology, shifting its timing and synchrony and decoupling plant and pollinator cycles (Clealand *et al.*, 2007; Memmott *et al.*, 2007; Parmesan and Hanley, 2015; Freimuth *et al.*, 2022). For example, the reproductive success of an individual plant might depend on flowering synchrony between conspecifics, which is essential for cross-pollination (Augspurger, 1981, 1983; Forrest and Miller-Rushing, 2010; Soares *et al.*, 2021), and on the degree of coupling with suitable environmental conditions for flowering (Puterill *et al.*, 2004; Kudo, 2006; Rodríguez-Pérez and Traveset, 2016; Culley *et al.*, 2020; Soares *et al.*, 2021). Therefore, it is important not only when the flowering begins or ends, but also how flowering is distributed over time (Thomson, 1980; Clark and Thompson, 2011). All these factors constitute selective forces shaping the flowering phenologies of plant populations and communities (Gómez, 1993; Elzinga *et al.*, 2007; Memmott *et al.*, 2007).

The timing of flowering phenology is influenced by a number of abiotic factors, including warm spring temperatures (forcing), cold winter temperatures (chilling), night–day length ratio (photoperiod), precipitation, elevation and other environmental cues (Imaizumi and Kay, 2006; Iannucci *et al.*, 2008; Giménez-Benavides *et al.*, 2010; Springate and Kover, 2014; Kharouba *et al.*, 2017; Flynn and Wolkovich, 2018). In recent decades, changes in the phenological patterns of species owing to climate change have been reported, particularly in the Northern Hemisphere (Menzel *et al.*, 2006; Kharouba *et al.*, 2017; United Nations Environment Programme, 2022; Vitasse *et al.*, 2022). The most notable effect of climate change is the increase in temperatures triggering an earlier onset of spring and an advance in the time of first flowering of species in the Northern Hemisphere (Menzel *et al.*, 2006; Schwartz *et al.*, 2006; Scheip *et al.*, 2009; Amano *et al.*, 2010; CaraDonna *et al.*, 2014; Szabó *et al.*, 2016; Zohner *et al.*, 2017; Vitasse *et al.*, 2022). Other environmental cues that plants use to time their flowering, such as precipitation, have also been affected by climate change and might be crucial in Mediterranean areas and tropics (Peñuelas *et al.*, 2002; Thuiller 2007; Calzadilla *et al.*, 2013; Chambers *et al.*, 2013). Despite this general trend, some studies have found that the effects of climate change are species specific, with some species flowering earlier or later than in the past, whereas others remain unaffected (Fitter and Fitter, 2002; Chambers *et al.*, 2013; CaraDonna *et al.*, 2014).

One of the consequences of the change in flowering time is the decoupling of plant phenologies, which has important implications for plant reproduction, ecosystem functioning (Totland and Alatalo, 2002; Kudo and Hirao, 2006; Forrest and Miller-Rushing, 2010; Iler *et al.*, 2019; Kudo and Cooper, 2019) and species conservation (Morellato *et al.*, 2016). For example, a change in flowering phenology can disrupt the synchrony between a plant population and its pollinators (Forrest and Thomson, 2011), potentially leading to decreased pollination success and reduced seed production (Memmott *et al.*, 2007; Morellato *et al.*, 2016). Furthermore, phenological shifts can have strong effects on community structures and ecological interactions between plant and animal species (Suttle *et al.*, 2007; Yang and Rudolf, 2010; Renner and Zohner, 2018; United Nations Environment Programme, 2022). Given that the flowering phenology of each plant species can show different responses to external factors (Rathcke and Lacey, 1985; Cortés‐Flores *et al.*, 2017; Renner and Zohner, 2018), climate change might affect the flowering phenology of each species in different ways. Plant species in a community establish competitive or facilitative interactions mediated by pollinators (Bergamo *et al.*, 2020); therefore, if the timing of their flowering events shifts, these interactions might be altered, affecting species differently (Albor *et al.*, 2020, 2022). As a result, the rearrangement of co-flowering patterns might limit the persistence of individual species in the community (Gilman *et al.*, 2010).

Despite the importance of identifying the causes and consequences of climate change on flowering phenology, the lack of past data to compare with contemporary information is a major limitation, especially in highly diverse ecosystems (Chambers *et al.*, 2013; Morellato *et al.*, 2016; Abernethy *et al.*, 2018). Many researchers use natural history collections (Calinger *et al.*, 2013; Aufret, 2021) or government data (Gordon and Sanz, 2009), but these provide information on changes only at the species or species group level (e.g. Davis *et al.*, 2015; Mathews and Mazer, 2016; Meineke *et al.*, 2018). Furthermore, much of the legacy data provides information only on the onset of flowering (first date), with very little information on complete flowering periods (CaraDonna *et al.*, 2014; Morellato *et al.*, 2016). However, the use of historical observations has been proved to be a reliable source of long-term data, uncovering trends and shifts of plant phenology across decades (Miller-Rushing and Primack, 2008; Miller *et al.*, 2021). Finally, information at the community level (Cook *et al.*, 2012; Ovaskainen *et al.*, 2013; CaraDonna *et al.*, 2014) or on the temporality of plant species interactions (Klanderud, 2005; Memmott *et al.*, 2007; Genini *et al.*, 2021) is still scarce, and plant–plant or plant–animal temporal associations are mostly ignored in models predicting how plant communities will respond to climate change.

The Mediterranean basin is one of the largest biodiversity hotspots of the planet (Mittermeier *et al.*, 2004), and abiotic constraints impose a strong seasonal limitation on flowering (Arroyo, 1990*a*, *b*; Petanidou *et al.*, 1995). The Mediterranean basin has been identified as one of the most vulnerable regions to the effects of climate change, owing to higher temperatures and more frequent droughts (Ali *et al.*, 2022), and its impact on floral phenology is expected to be strongly pronounced (Peñuelas *et al.*, 2004; del Cacho *et al.*, 2013). In this study, we compared the complete flowering phenology of 51 perennial species from a Mediterranean community 35 years ago (1985–87) with that obtained in the present (2020–22) for the same site and species. Our aims were as follows: (1) to verify the occurrence of shifts in the flowering periods after 35 years; (2) to detect climate drivers and changes over time; and (3) to uncover and analyse changes in co-flowering scenarios among Mediterranean species attributable to the expected phenological shifts.

## MATERIALS AND METHODS

### Study site and study species

Our study system was a rich Mediterranean plant community located in a lowland flat area at 80–90 m a.s.l., ~30 km from the sea, within the municipality of Hinojos, in the Huelva Province (37°15ʹ–37°20ʹN and 6°30ʹ–6°32ʹW), south-west Spain (Ortiz, 1991). The community consists of a mixed scrub and rich grassland with scattered to dense woodland, mainly *Quercus suber* and *Pinus pinea* (Ortiz, 1991). The shrub layer is diverse, with some of the better-represented families being Cistaceae, Lamiaceae and Fabaceae. The herbaceous layer is much more diverse, consisting mainly of species of Poaceae, Liliaceae, Boraginaceae, Plantaginaceae, Lamiaceae, Brassicaceae, Fabaceae, Caryophyllaceae and Asteraceae. We studied the flowering phenology of the 51 most abundant perennial species of the community, including small trees, shrubs and perennial herbs (Table 1) in two periods: 1985–1987 (hereafter, decade 1980s), with data collected by one of the authors (Ortiz, 1991); and present, 2020–2022 (hereafter, decade 2020s), re-collected for the same site and species.

**Table 1.** List of studied species, including family, type of life form, main pollination agent (based on personal records) and number of individuals sampled per studied year. Note that for some years we had information only on the start, peak or end of each flowering period.

				Year					
Species	Family	Type	Pollination agent	1985	1986	1987	2020	2021	2022
*Arbutus unedo* L.	Ericaceae	Shrub/tree	Small to big bees, hoverflies	–	20	21	–	13	17
*Aristolochia baetica* L.	Aristolochiaceae	Vine	Very small flies (Phoridae, etc.)	–	10	10^c^	–	22	20
*Salvia rosmarinus* Spenn.	Lamiaceae	Shrub	Small to big bees, hoverflies, butterflies	–	20	13^d^	–	16	18
*Ulex eriocladus* C. Vicioso	Fabaceae	Shrub	Medium to big bees, hoverflies	–	21	13^d^	–	26	24
*Halimium calycinum* K. Koch	Cistaceae	Suffrutix	Small bees, hoverflies, flies	–	12	12^d^	–	18	18
*Anchusa calcarea* Boiss.	Boraginaceae	Perennial/biennial herb	Small to big bees, hoverflies, beeflies, butterflies	–	11	–	–	–	15
*Stauracanthus genistoides* (Brot.) Samp.	Fabaceae	Shrub	Medium to big bees	–	12	–	–	15	15
*Lavandula stoechas* L.	Lamiaceae	Suffrutix	Small to big bees, hoverflies, beeflies, butterflies	–	12	12^c^	–	15	20
*Teucrium fruticans* L.	Lamiaceae	Shrub	Medium to big bees	–	12	12^c^	–	15	24
*Phyllirea angustifolia* L.	Oleaceae	Shrub	Wind	–	13	–	–	14	13
*Scrophularia frutescens* L.	Scrophulariaceae	Suffrutix	Small to big bees, hoverflies	–	12	–	–	16	20
*Genista triacanthos* Brot.	Fabaceae	Shrub/suffrutix	Medium to big bees	–	12	–	–	15	15
*Erica umbellata* Loefl. ex L.	Ericaceae	Shrub	Small bees	–	11	–	–	7	11
*Cytisus grandiflorus* (Brot.) DC.	Fabaceae	Shrub/tree	Small to big bees	–	14	–	–	13	13
*Echium gaditanum* Boiss.	Boraginaceae	Perennial/biennial herb	Small to big bees	–	11	–	–	–	20
*Crataegus monogyna* Jacq.	Rosaceae	Shrub/tree	Small to big bees, hoverflies	–	12	–	–	15	13
*Lysimachia monelli* (L.) U.Manns & Anderb.	Primulaceae	Perennial herb	Small bees, hoverflies	–	10	–	–	–	20
*Cistus albidus* L.	Cistaceae	Shrub	Small to big bees, hoverflies	–	11	–	–	15	30
*Rhamnus lycioides* L.	Rhamnaceae	Shrub	Small to big bees, hoverflies	–	12	–	–	11	17
*Cistus salviifolius* L.	Cistaceae	Shrub	Small to big bees, hoverflies	–	12	–	–	20	29
*Pistacia lentiscus* L.	Anacardiaceae	Shrub/tree	Wind	–	14	–	–	12	14
*Cistus ladanifer* L.	Cistaceae	Shrub	Small to big bees, hoverflies	–	12	–	–	20	30
*Osyris alba* L.	Santalaceae	Shrub	Hoverflies, flies	–	12	–	–	15	20
*Quercus coccifera* L.	Fagaceae	Shrub	Wind	–	12	–	–	–	14
*Coronilla juncea* L.	Fabaceae	Shrub	Small to big bees	–	13	–	–	15	15
*Genista hirsuta* Vahl	Fabaceae	Shrub/suffrutix	Medium to big bees	–	12	–	–	15	15
*Cistus monspeliensis* L.	Cistaceae	Shrub	Small to big bees, hoverflies	–	12	–	–	20	30
*Cistus libanotis* L.	Cistaceae	Shrub	Small to big bees, hoverflies	–	13	–	–	15	30
*Halimium halimifolium* (L.) Willk.	Cistaceae	Shrub/suffrutix	Small to big bees, hoverflies, beetles	–	12	–	15	23	30
*Armeria gaditana* Boiss.	Plumbaginaceae	Perennial herb	Small to big bees	–	12	–	–	15	18
*Erica scoparia* L.	Ericaceae	Shrub	Wind	–	12	–	–	12	12
*Phlomis purpurea* L.	Lamiaceae	Shrub	Big bees	–	11	–	–	12	15
*Cistus psilosepalus* Sweet	Cistaceae	Shrub	Small bees	–	12	–	–	13	20
*Cistus crispus* L.	Cistaceae	Shrub	Small to big bees, hoverflies, butterflies	–	12	–	–	15	30
*Anthyllis cytisoides* L.	Fabaceae	Shrub/suffrutix	Small to big bees	–	12	–	15	15	15
*Lonicera implexa* Aiton	Caprifoliaceae	Shrub/vine	Small to big bees, Lepidoptera	–	9	–	–	4	5
*Armeria velutina* Welw. ex Boiss. & Reut.	Plumbaginaceae	Perennial herb	Small to big bees, butterflies	–	12	–	–	15	18
*Retama sphaerocarpa* (L.) Boiss.	Fabaceae	Shrub/tree	Small to big bees, wasps, hoverflies	–	12	–	–	7	8
*Thymus mastichina* L.	Lamiaceae	Suffrutix	Small to big bees, wasps, butterflies	–	12	–	15	15	–
*Clematis flammula* L.	Ranunculaceae	Vine	Small to big bees	–	12	–	–	15	7
*Helichrysum stoechas* DC.	Asteraceae	Suffrutix/perennial herb	Small to big bees, beeflies, butterflies	–	12	–	–	16	15
*Rubus ulmifolius* Schott	Rosaceae	Shrub	Small bees, butterflies	–	12	–	10	7	10
*Myrtus communis* L.	Myrtaceae	Shrub	Small to big bees, beeflies	–	12	–	15	15	–
*Teucrium capitatum* L.	Lamiaceae	Suffrutix	Small bees, wasps	–	12	–	15	16	–
*Dianthus inoxianus* Gallego	Caryophyllaceae	Perennial herb	Small bees, sphingids	–	10	–	–	8	–
*Helichrysum picardii* Boiss. & Reut.	Asteraceae	Suffrutix/perennial herb	Small to big bees, beeflies, butterflies	–	12	–	15	–	–
*Daphne gnidium* L.	Thymelaeaceae	Shrub	Small to big bees, wasps, hoverflies, butterflies	20^a^	13	–	15	14	–
*Carlina corymbosa* L.	Asteraceae	Perennial herb	Small to big bees, beeflies	–	12	–	–	18	–
*Dittrichia viscosa* (L.) Greuter	Asteraceae	Perennial herb	Small bees, hoverflies	20^b^	12	–	–	5	–
*Smilax aspera* L.	Smilacaceae	Liana	Hoverflies, flies	18	13	–	12	14	–
*Calluna vulgaris* (L.) Hull	Ericaceae	Shrub	Small to big bees, hoverflies	–	12	–	–	18	–

^a^Data for only the end of the flowering period available.

^b^Data for only the peak and the end of the flowering period available.

^c^Data for only the start of the flowering period available.

^d^Data for only the start and the peak of the flowering period available.

### Climate

The climate in the study area is typically Mediterranean, with hot, dry summers and mild winters (Ortiz, 1991; Deitch, *et al.*, 2017). Climatic variables from 1974 to 2022 were provided by the Andalusian Environmental Information Network (REDIAM). We selected a data series from three nearby climate stations (<34 km from study sites; Supplementary Data Table S1). The data from the three stations were similar enough for us to average them in order to minimize data gaps throughout the studied period. The climatic variables obtained were daily mean temperature, daily minimum temperature, daily maximum temperature and daily rainfall. For temperature data, we obtained the annual means and the means for the four meteorological seasons (winter: Dec, Jan, Feb; spring: Mar, Apr, May; summer: Jun, Jul, Aug; autumn: Sep, Oct, Nov) during the periods of study. For precipitation data, we obtained the annual accumulated rainfall and the accumulated rainfall for the four meteorological seasons during the periods of study.

### Flowering phenology in 1980s and 2020s

From August 1985 to May 1987, weekly flower counts were made on 9–21 individuals for each of the 51 species studied in order to assess their annual flower production (Ortiz, 1991); we now use the raw data obtained in those counts to study flowering phenology in that decade. Thirty-six years later, to assess possible phenological changes, we reproduced the same type of counts on 4–30 individuals of the same species from April 2020 to July 2022 (Table 1).

We pooled all data for each sampling decade and species, generating two datasets for each species: 1980s and 2020s. For each decade and species, we obtained the mean start date of flowering, the mean end date of flowering, the flowering peak (as the median for the date of highest flower production) and the mean duration of the flowering period.

### Changes in co-flowering patterns

In order to determine whether co-flowering patterns between species in the community had changed, we created a co-flowering matrix for each of the study decades, the 1980s and the 2020s. In each matrix, co-flowering for each focal species with each other species in the community was calculated as the number of overlapping days divided by the total number of flowering days of the focal species. These matrices showed the proportion of days that each focal species overlapped with other species in each decade. Then, we obtained a co-flowering change matrix showing the increase or decrease in overlap between species by dividing the values for the 2020s by those for the 1980s. In this matrix, if the proportion of co-flowering of species ‘A’ with species ‘B’ had changed from 0.2 to 0.4, the value of the cell would be 2, whereas a change from 0.4 to 0.2 would give a value of −0.5. If a species gained a new co-flowering neighbour from the 1980s to the 2020s, the cell would be marked as a ‘gain’, whereas if a co-flowering interaction was lost, the cell would be marked as a ‘loss’.

Then, in order to address specifically how changes in co-flowering affected species that shared pollinators, we combined the co-flowering change matrix with the information on main pollinators for each species. Initially, we reduced the co-flowering change matrix to a matrix with only three possible values: increased co-flowering (1), decreased co-flowering (−1) and unaffected co-flowering (0); then, we selected only the combinations of species that had changes in co-flowering and shared their main pollinators (Table 1), thus being potential competitors for their attention (Mitchell *et al.*, 2009); finally, we calculated the sum of the values for each species, the total sum for all the species and the mean value.

### Statistical analysis

In order to determine the magnitude and rate of change of the climatic variables in the study area, linear models were fitted, with the climatic variable of interest as the dependent variable and time as the independent variable. These models were performed using both daily and annual data. Additionally, we performed the same models considering exclusively data for each meteorological season.

Given that phenological phenomena follow a repetitive cycle, in which each year is an iteration of the cycle, we used circular statistics to deal with date analyses (Morellato *et al.*, 2000, 2010, 2016). Following Morellato *et al.* (2000, 2010), we transformed all dates into angular degrees. Then, we tested flowering dates in both decades for unimodality and significance of mean angle (or mean dates) of flowering by using the Rayleigh test (Morellato *et al.*, 2010). If significant, the pattern is considered significantly seasonal, and the vector *r* is a measure of the degree of seasonality of phenological dates (Morellato *et al.*, 2000). The value of *r* ranges from zero (no seasonality and a uniform distribution) to one (the highest seasonality) (Morellato *et al.* 2000, 2010). In order to test whether the significant mean angle (or mean date) of the community had changed from the 1980s to the 2020s, we tested whether the number of species flowering each day of the year between the two decades differed by performing the Watson–Williams *F*-test. In order to test whether the degree of seasonality had changed from the 1980s to the 2020s, we performed an equal kappa test, which compares dispersion of the circular data. We also tested whether the mean angular start and mean end dates of flowering differed from the 1980s to the 2020s, using again the Watson–Williams *F*-test. Additionally, we tested for changes in the date of maximum flower production using the Wilcoxon test. The Rayleigh test, the Watson–Williams *F*-test and the equal kappa test were implemented using the ‘circular’ package (Agostinelli and Lund, 2022), whereas the Wilcoxon test was implemented using the ‘TwoCircles’ package (Guerrier and Jammalamadaka, 2018), all in the R statistical environment (R Core Team, 2022).

In order to test whether certain lineages are more susceptible to climate change-induced shifts in flowering phenology, we estimated the phylogenetic signal in two of our variables (flowering start shift and flowering duration change) by calculating Pagel’s λ (Pagel, 1999) and estimating its significance with the the ‘phytools’ package (Revell, 2012). We obtained a phylogenetic tree for our data by trimming the tree ‘GBOTB.extended’ to our species list with the ‘V.Phylo.Maker’ package (Qian and Jin, 2016). The mentioned tree is available within the same package, and all phylogenetic analyses were performed in the R statistical environment (R Core Team, 2022). Of our 51 studied species, two (*Retama sphaerocarpa* and *Stauracanthus genistoides*) were not in the tree and could not be included in the phylogenetic signal analyses. Nevertheless, the Fabaceae family is well represented in our dataset, and these species did not show extreme values of phenological change; therefore, we expect that the exclusion of these two species would not affect the results of phylogenetic signal analyses.

In order to test whether the duration of the flowering period changed between decades, we used an ANOVA. We also fitted a linear model, with flowering period duration as the dependent variable and the flowering start date as the independent variable, to test whether an earlier flowering start was related to an increase in flowering duration. Both the ANOVAs and linear models were performed as implemented in the ‘stats’ package (R Core Team, 2022).

We used the ‘lubridate’ package (Grolemund and Wickham, 2011) and the ‘ggplot2’ package (Wickham, 2016) to produce plots in the R statistical environment.

## RESULTS

### Changes in climatic variables

**Fig. 1.**
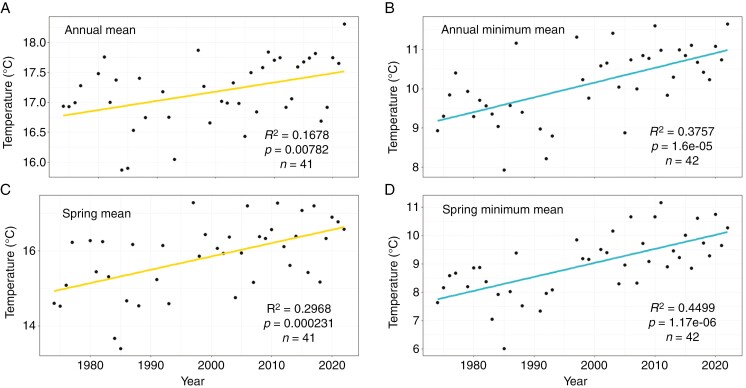
Patterns of temperatures and fitted linear models for the study area of Hinojos, in the Huelva Province, south-west Spain. (A) Annual mean temperatures. (B) Annual mean minimum temperatures. (C) Spring mean temperatures. (D) Spring mean minimum temperatures.

The mean annual temperature increased from 16.93 °C in 1974 to 17.52 °C in 2022 (Fig. 1A; Supplementary Data Fig. S1A). Likewise, the mean annual minimum temperature also increased from 8.93 °C in 1974 to 11.65 °C in 2022 Fig. 1B; Supplementary Data Fig. S1B), and the mean annual maximum temperature also increased from 24.93 °C in 1974 to 25.86 °C in 2022 (Supplementary Data Fig. S1C). Linear regression models were statistically significant for mean annual temperatures (Fig. 1A) and for minimum annual temperatures (Fig. 1B), in both cases showing an increasing trend. Temperature changes were not uniform across all seasons: for seasonally averaged data models, only spring mean temperatures increased significantly (Fig. 1C; Supplementary Data Fig. S2), spring, summer and autumn mean minimum temperatures increased significantly (Fig. 1D; Supplementary Data Fig. S3), and again only spring mean maximum temperatures showed a significant increase (Supplementary Data Fig. S4). The spring minimum temperature model equation showed the highest coefficient, which translates into an increase of almost 0.5 °C every 10 years (Fig. 1D).

The annual accumulated precipitation showed a decrease from 520 mm in 1974 to 290 mm in 2022 (Supplementary Data Fig. S1D), but the linear models were not significant. The seasonally averaged accumulated precipitation models were also not significant (Supplementary Data Fig. S5).

### Flowering phenology

**Fig. 2.**
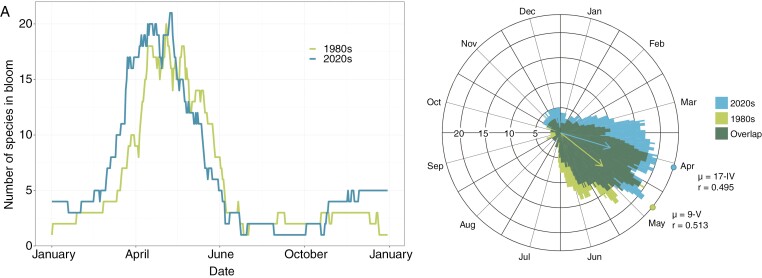
Number of plant species flowering in the Mediterranean shrub community throughout the year in the decades of the 1980s and 2020s. (A) Linear patterns highlighting the early spring flowering in 2020. (B) Circular distribution, with the arrow indicating the direction pointing to the angular mean date (*μ*); the arrow length is proportional to data concentration (*r*).

In the two studied decades, flowering phenology at the community level was highly seasonal (*P* < 0.05), with a high number of species flowering from late winter to early summer (Fig. 2A). The degree of seasonality did not change significantly between decades (Fig. 2B). However, the mean flowering peak of the community shifted significantly to an earlier date, from 9 May in the 1980s decade to 17 April in the 2020s decade (22 days). The maximum number of species flowering at the same date was ~20 in both decades (Fig. 2). The distribution of the community flowering curve differed between decades: in the 1980s, the period with ≥10 species in flower, about half of the maximum, lasted from 28 March to 25 June, whereas in the 2020s this period lasted from 12 March to 13 June (Fig. 2). There was also a decrease in the number of flowering species in the summer from the 1980s to the 2020s (Fig. 2).

**Fig. 3.**
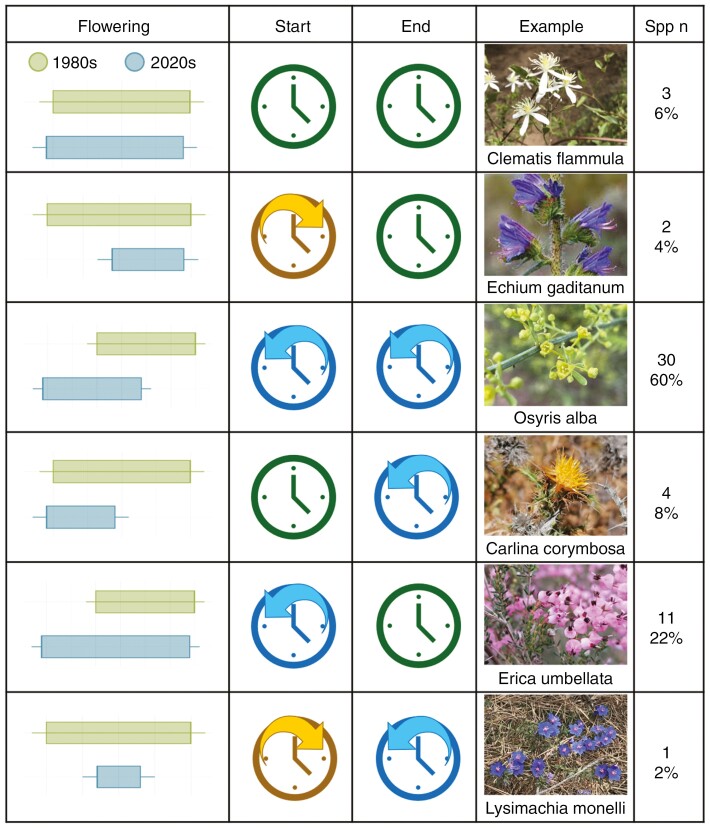
Patterns of change in start and end of flowering in the Mediterranean shrub community studied. The ‘Flowering’ column shows a representation of the different types of changes in the flowering period from the 1980s decade to the 2020s. The ‘Start’ and ‘End’ columns show the direction of change for the flowering start and end dates, respectively (green clocks = no changes; yellow clocks with a right-pointing arrow = change towards a later date; and blue clocks with a left-pointing arrow = change towards an earlier date). The ‘Example’ column provides a picture and name of a species representing each type of phenological change. The ‘Spp n’ column shows the number and percentage of species presenting each type of phenological change.

Considering phenological shifts at the species level between the 1980s and 2020s decades, 80 % of the species showed a significant (*P* < 0.05) advancement in their flowering start date, and only 6 % showed a significant delay (Fig. 3; Supplementary Data Table S2). Taking into account only the species that flowered earlier, the advance in the start date ranged from 6 to 92 days (mean 24 days in advance; Supplementary Data Table S2). The date of the end of the flowering also showed a significant advancement in 68 % of the species between decades, and none of the species had a significant delay (Supplementary Data Table S2). Regarding combinations of changes in both start and end flowering dates (Fig. 3), 59 % of species showed a shift to earlier start and end dates, resulting in a complete advancement of their flowering period. Meanwhile, 21 % of species showed an advancement only in the start date, and 8 % only in the end date (Fig. 3). Only 6 % of the studied species did not show a significant shift of their flowering start or end dates, and 4 % showed a later start date but the end date remained unchanged. Only one species (*Lysimachia monelli*) showed a delay in its flowering start date and an advancement of its flowering end date, resulting in a reduction of its total flowering period (Fig. 3). Shifts were unevenly distributed across species; those that flowered at earlier dates, such as *Salvia rosmarinus*, *Aristolochia baetica* and *Ulex eriocladus*, advanced more than average, whereas the ones flowering at later dates, such as *Clematis flammula*, *Dianthus inoxianus* and *Calluna vulgaris*, advanced their flowering period little or not at all.

Regarding changes in the distribution of the flowering peak date, 90 % of the species showed a significant difference between decades (Supplementary Data Table S2). Of all the species, 82 % showed an advancement of their median date, whereas 6 % showed a delay. One species (*Daphne gnidium*) showed differences in its flowering peak date distribution, but its median date did not change from the 1980s to the 2020s; instead, its distribution became wider.

The length of the flowering period showed a significant increase in 43 % of the species (*P* < 0.05), whereas 20 % of the species showed a significant decrease and 37 % showed no change between decades (Supplementary Data Table S2).

**Fig. 4.**
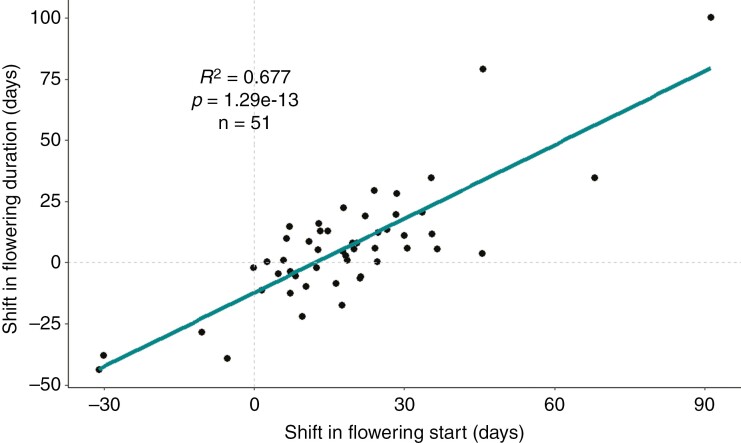
Shifts in flowering duration (in days) in relationship to the shift in flowering start date (in days) from the decade of the 1980s to the decade of the 2020s for the 51 species studied in the Mediterranean shrub community. Points are raw data (*n* = 51), and the line represents predicted values from the linear regression model.

There was no phylogenetic signal for either of the two analysed variables [flowering start date (λ = 6.64325 × 10^−5^, *P* = 1; Supplementary Data Fig. S6A) or change in flowering duration (λ = 5.98388 × 10^−5^, *P* = 1; Supplementary Data Fig. S6B), suggesting that species underwent phenological changes regardless of their evolutionary relationship. There was a positive and significant association between the change in the length of the flowering period and the advancement of flowering start date (Fig. 4), meaning that species that suffered a stronger shift in their start of flowering to an earlier date also showed an increased flowering duration.

### Changes in flowering order and co-flowering patterns

**Fig. 5.**
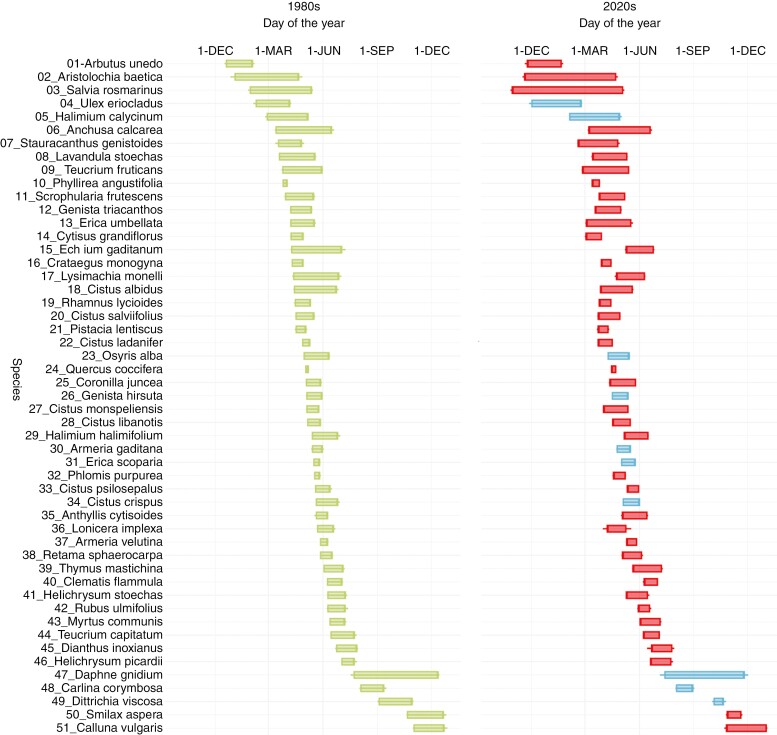
Flowering of the 51 studied species in the decades of the 1980s and 2020s in the Mediterranean shrub community. Species are ordered from top to bottom according to the date when they started flowering in the 1980s. Bars span from the mean start date to the mean end date for each species; lines show the standard deviation. Legend: Green bars = 1980s phenology; Blue and red bars = 2020s phenology, blue bars = unchanged ranking order, red bars = changed ranking order.

Phenological rank order within the community changed from the 1980s decade to the 2020s decade (Fig. 5; Supplementary Data Table S3). Although most species underwent only a small change in their flowering rank order, some shifted considerably. For instance, *Echium gaditanum* shifted from being the 15th to the 36th species to start flowering, and *Lonicera implexa* shifted from the 36th to the 22nd.

**Fig. 6.**
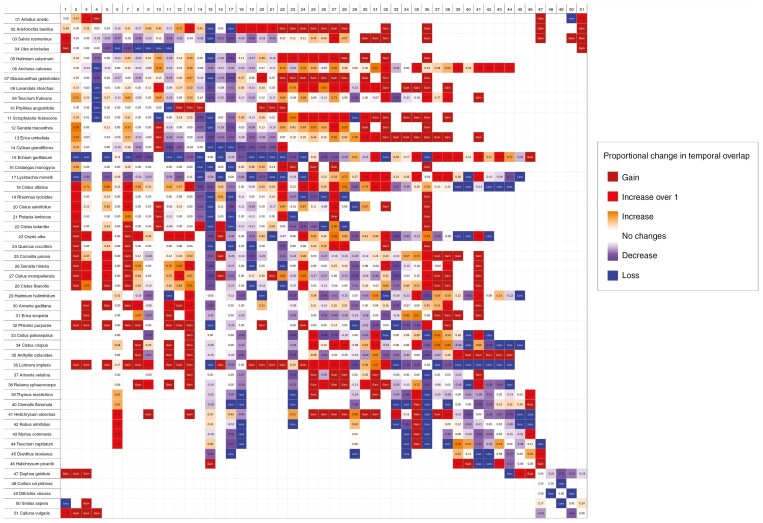
Community-level matrix of change in co-flowering overlap in our Mediterranean shrub community. Each cell represents the proportional change in co-flowering overlap between species pairs in the two study decades of the 1980s and 2020s, 35 years apart. To represent the change in co-flowering visually for all species, we divided the co-flowering proportion value of the 2020s matrix by the value in the 1980s matrix (see Supplementary Data Figs S7, S8); thus, if the proportion of co-flowering of species ‘A’ with species ‘B’ had changed from 0.2 to 0.4, the value of the cell would be 2, whereas a change from 0.4 to 0.2 would give a value of −0.5. If a species gained a new co-flowering neighbour from the 1980s to the 2020s, the cell would be marked as a ‘gain’, whereas if a co-flowering interaction was lost, the cell would be marked as a ‘loss’. Cells are coloured to emphasize the message, hence a deeper shade of orange means an increase in co-flowering overlap, whereas a deeper shade of purple means a decrease in co-flowering overlap. Net gains and losses of co-flowering overlap were coloured as red and blue, respectively, in order to differentiate them.

Regarding co-flowering patterns, the matrix of co-flowering changed drastically from the 1980s (Supplementary Data Fig. S7) to the 2020s (Supplementary Data Fig. S8). A total of 324 co-flowering interactions in the 1980s increased, including the gain of 102 completely new co-flowering interactions in the 2020s (Fig. 6). Meanwhile, 252 co-flowering interactions decreased, including the complete loss of 74 co-flowering interactions (Fig. 6).

In the 1980s decade, 38 % of species shared at least part of their flowering period with more than half of the community (Supplementary Data Fig. S7). *Anchusa calcarea* was the species sharing its flowering period with the most neighbours (84 %), whereas *Carlina corymbosa* was the species sharing its flowering period with the fewest neighbours (only two species). Meanwhile, in the 2020s decade, 54 % of species shared at least part of their flowering period with more than half of the community (Supplementary Data Fig. S8). *Anchusa calcarea* was again the species with the most co-flowering neighbours (82 %), and both *Carlina corymbosa* and *Dittrichia viscosa* were tied as the species with the fewest co-flowering neighbours (only one species).

The matrix of changes in co-flowering for the species that shared their main pollinators (Supplementary Data Fig. S9) revealed important differences between species. Four species were left out of the analysis, because they were wind pollinated (Table 1). Only one entomophilous species (*Aristolochia baetica*) did not share its main pollinators with any other. Most entomophilous species (26, i.e. 55 %) increased their co-flowering with potential competitors; 18 of them (38 %) decreased their co-flowering with potential competitors, and 3 of them (6 %; including *A. baetica*) showed no overall increase or decrease. The species with the highest positive value for increased co-flowering with potential competitors was *Scrophularia frutescens* (14), whereas the species with the greatest decrease in co-flowering with potential competitors were *Cytisus grandiflorus* and *Halimium halimifolium* (both −9). The mean value of changes in co-flowering with potential competitors was 1.17 ± 5.15, but the total sum of the values was 55, indicating that the overall co-flowering with potential competitors remained relatively stable, although some species had extreme positive values.

## DISCUSSION

We demonstrated that Mediterranean plant communities and perennial species have shifted their overall flowering phenology since the decade of the 1980s, in terms of both start and end dates and duration. These shifts followed the increases in temperature related to climate change (IPCC, 2021) and were more pronounced for species that historically flowered in winter or early spring, and for the start and peak flowering dates. As a result of the different rates at which species were shifting, co-flowering neighbours changed between decades, with new co-flowering species pairs and novel ecological interactions. The new arrangements suggest that competitive or facilitative interactions between plant species might be emerging.

### Climate change and flowering phenology

Climate change projections over the Mediterranean region show a marked increase in temperatures and reduction in precipitation for the south-west Mediterranean (Giorgi and Lionello, 2008). These trends might be important in shifting the climate zone from mediterranean to arid by the end of the century (Alessandri *et al.*, 2014). Accordingly, the Mediterranean region has been described as a climate change ‘hot spot’ (Giorgi and Lionello, 2008; Lionello and Scarascia, 2020). Our results are in line with these predictions, because we found a significant increase in mean annual temperatures from the late 1970s to the present. Warming in our study area has occurred all year round, with a mean increase of 0.59 °C over the last 50 years, but being much stronger in the spring (1.8 °C). According to the latest Intergovernamental Pannel of Climate Change (IPCC) report, the projected mean annual warming in the Mediterranean basin by the end of the century will be in the range of 0.9–5.6 °C compared with the last two decades of the 20th century, depending on the emission scenario (IPCC, 2021). Our study confirmed an earlier and warmer spring in our study area, contrasting with predictions for the Mediterranean basin, where warming is expected to be much more pronounced in autumn and especially in summer (Lionello and Scarascia, 2018).

The warming in our study area was stronger for the minimum (≤2.7 °C) than for maximum daily temperature, determining a decrease in the amplitude of the daily temperature range, although some models have predicted the opposite pattern for the Mediterranean region (Lionello and Scarascia, 2018, 2020). Asymmetric differences in day/night temperatures reported here are found in the Northern Hemisphere (Solomon *et al.*, 2007). Daytime and nighttime temperature might influence plant phenology unequally (Wang *et al.*, 2019). Thus, observed shifts in flowering phenology might be more sensitive to variations in minimum temperatures in the season preceding flowering than to maximum temperatures. Daily temperature ranges are reported to influence plant flowering through effects on plant metabolism and development and on pollination (Peng *et al.*, 2013; Wang *et al.*, 2019).

We found a decrease of annual accumulated rainfall from 1975 to 2022 of ~25 %, which was marginally non-significant, probably owing to the high interannual variability in precipitation, which is characteristic of the Mediterranean region (Deitch *et al.*, 2017). The latest report by the IPCC also predicts a decrease in precipitation across the Mediterranean basin by the end of the century (by 4–22 %, depending on the emission scenario; IPCC, 2021). Mediterranean communities are strongly seasonal (Ackerly *et al.*, 2014), which is conditioned by the distribution of temperatures and precipitation. In our study area, we found strong flowering seasonality that did not change over the last 35 years. However, we did find a considerable shift in the flowering phenology of the community, because the community-scale flowering peak advanced >20 days since the 1980s. This shift is concurrent with the increase of spring temperatures; as spring gets warmer, the anticipated warming temperatures trigger an early flowering (Menzel *et al.*, 2006; Visser *et al.*, 2022).

We demonstrated that most species shifted towards earlier dates not only at the start but also at the anticipated end of the flowering period. Additionally, many species shifted to either an earlier start or end of the flowering period, all of which led to an advancement of the entire flowering period, a new and underexplored aspect of phenological responses to warming (Fig. 4). The early flowering shifts were pervasive in the community and in line with the trends described for first flowering for Northern Europe (Menzel *et al.*, 2020). Only five species did not experience a shift in either the start or the end of their flowering period.

Nevertheless, the advance in flowering phenology was of an unprecedented magnitude in our plant community. We found a mean advance in the beginning of the flowering of 21 days in a 35-year period (0.6 days year^−1^), 3-fold higher than previously described, and a maximum advance value of 90 days. For instance, in the Mediterranean, Peñuelas *et al.*, (2002) describe a mean advance in the beginning of flowering of 9.5 days from 1952 to 2000 (0.2 days year^−1^) and a maximum advance value of 70 days. Spring temperatures were the climatic variable that showed the greatest increase in our lowland Mediterranean study area and were significantly related to flowering. Therefore, we concluded that temperature must have played a key role in shifting the flowering times of our community. Crimmins *et al.* (2010) observed that, in semi-arid, lowland ecosystems at Mt. Kimball (AZ, USA), the phenological advance of flowering phenology was influenced by the increase in spring temperatures (spring forcing), which had a strong effect on the advancement of the first flowering day.

Despite this strong general trend, shifts in flowering also varied in magnitude among our Mediterranean species, with some showing no shift at all. Flowering species-specific shifts are pointed out in a few studies (Peñuelas *et al.*, 2004; Crimmins *et al.*, 2010; Cook *et al.*, 2012; CaraDonna *et al.*, 2014; Theobald *et al.*, 2017), mostly regarding starting dates. Shifting responses of flowering phenology are generally reported as being stronger in annuals than in perennial plants (Petanidou *et al.*, 2014; Büntgen *et al.*, 2022). In contrast, annual plant phenologies are also very sensitive to rainfall in comparison to perennials (Crimmins *et al.*, 2010), and a reduction in autumn rainfall could lead to a delay in the phenology response of the annual plant community. Thus, the phenological shifts of the whole community are expected to follow more complex patterns when including other life forms aside from perennial plants, as in the present study.

Species that flowered in late winter during the 1980s decade showed the highest values of advance and extended flowering periods: *Arbutus unedo*, *Aristolochia baetica*, *Salvia rosmarinus* and *Ulex eriocladu*s all started flowering between late December and early February in the 1980s and all showed the greatest advances in mean start dates (between 26 and 92 days). Increased spring forcing has been shown to advance flowering phenology, whereas reduced winter chilling has the opposite effect (Flynn and Wolkovich, 2018). The net shift in phenology might depend on the particular sensitivity of plant species to these two effects, among other factors (Flynn and Wolkovich, 2018). It is likely that chilling does not play a role in the early flowering responses of species in our study area, because the species in the 2020s decade were flowering before the onset of winter. In fact, several studies have shown that some Mediterranean taxa (e.g. *Quercus*, *Olea*) do not require accumulated chilling hours to initiate flowering (Osborne *et al.*, 2000; Pinto *et al.*, 2011). In contrast, the accumulation of heat units has been reported to be a key factor influencing the phenology of Mediterranean vegetation (Spano *et al.*, 2013). We postulate that perhaps one reason behind the extreme shift in flowering time is precisely the absence of a chilling cue balancing out the effect of spring forcing in the lowland Mediterranean region studied. In contrast, although rainfall has decreased only slightly during the period studied, the observed increase in temperature must have led to a significant loss of soil water availability (Komuscu *et al.*, 1998), which would bring forwards the critical summer drought period. Several studies have shown that soil water availability affects Mediterranean species by bringing forwards their flowering when conditions become drier (Spano *et al.*, 2013). Therefore, the combined effect of higher temperatures and lower water availability would also be responsible for the strong phenological shift observed here; this would particularly affect spring-flowering species, which would escape the advancing soil drought with this phenological shift. In any case, sensitivity to water stress is highly variable among Mediterranean species (Spano *et al.*, 2013) and this will condition the strength of the response of each species.

In contrast, species that flowered in summer–autumn in the 1980s decade showed the smallest shifts or even no flowering shifts (*Dianthus inoxianus*, *Daphne gnidium*, *Carlina corymbosa* or *Smilax aspera*) (see Dunne *et al.*, 2003). We propose alternative and non-exclusive explanations: the late-blooming species could be equally sensitive to both chilling and forcing and, therefore, the effects of reduced chilling and increased forcing could be balancing each other (Flynn and Wolkovich, 2018). Cook *et al.* (2012) found that warm temperatures during the vernalization period (typically autumn and winter) can delay dormancy or the fulfilment of chilling requirements, thereby delaying spring events, such as flowering. Alternatively, given that photoperiod is a third factor, along with chilling and forcing, usually involved in determining flowering date (Wang *et al.*, 2020a), it is possible that late-blooming species might be strongly photoperiod dependent for flowering.

Our study clearly showed a consistent pattern of advancements in the start flowering date driving the increase in flowering duration. Only a few occasional differences in flowering duration have been associated with shifts in phenology (CaraDonna *et al.*, 2014), highlighting the relevance of detailed phenological observations covering the complete flowering event.

Our study is, to our knowledge, the first to analyse the phylogenetic signal of changes in phenological variables, rather than the phenological variables (dates and durations) themselves. We found no significant phylogenetic signal for the change of the phenological variables, which would indicate that the effects of climate change on the flowering phenology of our community are pervasive and more influenced by factors other than phylogeny (i.e. flowering season). Previous studies have shown that phylogeny is relatively good at predicting flowering phenology on a global scale (Davies *et al.*, 2013), but studies focusing on ecological communities have found a lack of phylogenetic signal for flowering phenology (Cortés‐Flores *et al.*, 2017; Wang *et al.*, 2020b), perhaps because the pool of species belonging to a community is already filtered by an underlying evolutionary history.

We have found a strong phenological reassembly, including the gain and the disappearance of co-flowering partners, and also a change in flowering order, probably related to climate change, for the first time in the studied Mediterranean community. Regarding co-flowering, the number of autumn- to winter-flowering species has doubled, whereas the number of summer-flowering species has decreased. Flowering reassembly occurs when changes in phenological patterns are species specific (Crimmins *et al.*, 2010), as detected in our community. Studies examining how climate reshapes co-flowering relationships at the community level are still very scarce (Mitchell *et al.*, 2009; Forrest *et al.*, 2010; Caradonna *et al.*, 2014; Faust and Iler, 2021), but they are crucial because such shifts influence the nature of ecological interactions that are shaped by the community context (Theobald *et al.*, 2017).

Additionally, there was an impressive change in the flowering order between the 1980s and the 2020s decades. The reshuffling of species in flower might affect the visits of pollinators to the plants, adding to the temporal effects of flowering anticipation and duration. The reassembly of flowering order related to climate change is an unexplored theme in climate change research, and we need to explore how pervasive the phenomenon is across landscapes and phenophases. However, flowering order is a relevant issue for pollination research and defines pollination patterns, such as the trapline visits by hummingbirds and bats, across time and space (Gentry, 1974; Lau *et al.*, 2017).

Although our data do not allow us to determine the consequences of temporal changes and flowering decoupling, phenological reassembly has the potential ultimately to impact the fitness of species through their relationships with pollinators (Lázaro *et al.*, 2009; Fantinato *et al.*, 2018) and pathogens (O’Brien *et al.*, 2021). For plant species, matching their flowering time and synchrony with pollinator activity is crucial for fitness (Augspurger, 1981; Labonté *et al.*, 2022), particularly for those species that are self-incompatible. That is the case for many of the studied Mediterranean species, because they are entomophilous and self-incompatible (e.g. *Cistus* and *Halimium* spp., *Lysimachia monelli*, *Phlomis purpurea*, *Salvia rosmarinus* or *Ulex eriocladus*) (Talavera *et al.*, 1997; Gibbs and Talavera, 2001; Magrach *et al.*, 2021). Co-flowering species frequently share pollinators and can compete for their services (Mitchell *et al.*, 2009; Johnson *et al.*, 2022), but co-flowering species can also be mutually beneficial if a higher number of open flowers in the immediate area facilitate pollinator attraction (Bergamo *et al.*, 2020). In this regard, most of our studied species are pollinated by bees or hoverflies, and we found a slight tendency towards increased co-flowering with other plants sharing pollinators, which might lead to a subsequent increase in competition or facilitation. Given that the number of species flowering varies widely throughout the year, there is the possibility that, at different times of the year, either competition or facilitation is prevalent at a community scale.

An example of the temporal decoupling between species sharing pollinators in our study area is *Cistus*, an important genus in terms of number of species (seven) and abundance, providing resources for a variety of pollinators and being self-incompatible (Bosch, 1992; Talavera *et al.*, 1993; Ortiz, 1994; Magrach *et al.*, 2021). We found a decrease in flowering overlap of *Cistus* species in the 2020s compared with the 1980s. Phylogenetic relatedness has been shown to mediate the effect of heterospecific pollen on post-pollination success, with possible consequences for reproduction (Streher *et al.*, 2020), and all *Cistus* species share their general main pollinator groups. If the *Cistus* species were mutually facilitating, the reduced flowering overlap could lead to a decrease in their current fitness, but if their relationships were competitive, we might expect an increase in the fitness of some species. According to Theobald *et al.* (2017), phenological reassembly can have a high impact on long-lived species, such as those studied here, because changes in abundance and distribution are slow relative to climate change, and this might have implications for species conservation (Morellato *et al.*, 2016).

In contrast, changes in the flowering phenology of the studied community might have important consequences from the pollinator point of view. In the Mediterranean basin, the most important pollinators are insects, and their activity is highly seasonal (Herrera, 1988; Petanidou *et al.*, 1995). In our study area, in the 1980s, pollinator activity was concentrated between March and June, depending on the taxonomic group (Herrera, 1988). This seasonal pattern suggests that pollinators would be very sensitive to warming and consequent changes in flowering phenology (Manincor *et al.*, 2023). If the pace in phenological shift differs between plants and pollinators, an ecological mismatch will result (Memmott *et al.*, 2007). Although some broad studies suggest that the phenological shift of flowering and pollinator activity might occur at roughly the same pace (Bartomeus *et al.*, 2011), other more specific studies have found differences in the shift of plants and their pollinators, resulting in some degree of mismatch (Kehrberger and Holzschuh, 2019; Kudo and Cooper, 2019; Manincor *et al.*, 2023). Owing to the generalization of ecological interactions in pollination networks, ecological mismatches of plants and pollinators might not have major negative consequences for most species (Hegland *et al.*, 2009). However, for the specialized species, the consequences might be dire (Memmott *et al.*, 2007; Hegland *et al.*, 2009). Furthermore, there is currently a need for more empirical evidence on ecological mismatch and its consequences at the community level (Gérard *et al.*, 2020; Iler *et al.*, 2021).

Thanks to the research produced or started in the 20th century, along with the access to legacy datasets (e.g. Miller-Rushing and Primack, 2008), we are beginning to understand the effects of climate change on important biological processes, such as the flowering of plants. However, there are very few studies providing evidence of the effect of climate change on emerging ecological interactions, as we have already pointed out, and on the evolutionary mechanisms by which plants can adapt their phenology to environmental changes. Among these mechanisms are genetic change under natural selection and phenotypic plasticity. Both mechanisms have been demonstrated to play a role in the phenological response of flowering to climate change (Anderson *et al.*, 2012; Richardson *et al.*, 2017). However, because perennials have longer life cycles, their response to selection must be slower (Yue *et al.*, 2010), and phenotypic plasticity is therefore likely to play a more important role in the phenological shifts observed in the shrub community studied (Yue *et al.*, 2010; and see Satake *et al.*, 2022). Further studies regarding the ecological and evolutionary processes governing plant phenological responses to the ongoing climate change remain necessary.

## SUPPLEMENTARY DATA

Supplementary data are available at *Annals of Botany* online and consist of the following.

Figure S1: evolution of climatic variables and fitted linear models for our study area. Figure S2: evolution of mean temperatures and fitted linear models for our study area. Figure S3: evolution of mean minimum temperatures and fitted linear models for our study area. Figure S4: evolution of mean maximum temperatures and fitted linear models for our study area. Figure S5: evolution of cumulative precipitation and fitted linear models for our study area. Figure S6: phylogenetic tree for our study species showing the phylogenetic signal inferred from shifts in flowering start date [log(number of days)] from the decade of the 1980s to the decade of the 2020s. Figure S7: community-level co-flowering overlap in the 1980s decade. Figure S8: community-level co-flowering overlap in the 2020s decade. Figure S9: community-level matrix of change in co-flowering overlap for the species that shared their main pollinators (as defined in Table 1). Table S1: list of meteorological stations used in this study. Table S2: list of studied species, including information on each studied phenological parameter for both decades, the direction of change, the significance level of the test (n.s. = non-significant, *P < 0.05, **P < 0.01) and the magnitude of the change. Table S3: list of studied species, including the order of the flowering start date in the 1980s decade and the 2020s decade.

mcad193_suppl_Supplementary_Tables_S1-S3

mcad193_suppl_Supplementary_Figures_S1

mcad193_suppl_Supplementary_Figures_S2

mcad193_suppl_Supplementary_Figures_S3

mcad193_suppl_Supplementary_Figures_S4

mcad193_suppl_Supplementary_Figures_S5

mcad193_suppl_Supplementary_Figures_S6

mcad193_suppl_Supplementary_Figures_S7

mcad193_suppl_Supplementary_Figures_S8

mcad193_suppl_Supplementary_Figures_S9
