# Supplementary figures and images for: Better soon than never: climate change induces strong phenological reassembly in the flowering of a Mediterranean shrub community
Source: Ann Bot. 2023 Dec 14;135(1-2):239–54. doi: 10.1093/aob/mcad193 (PMC11805945; doi:10.1093/aob/mcad193)

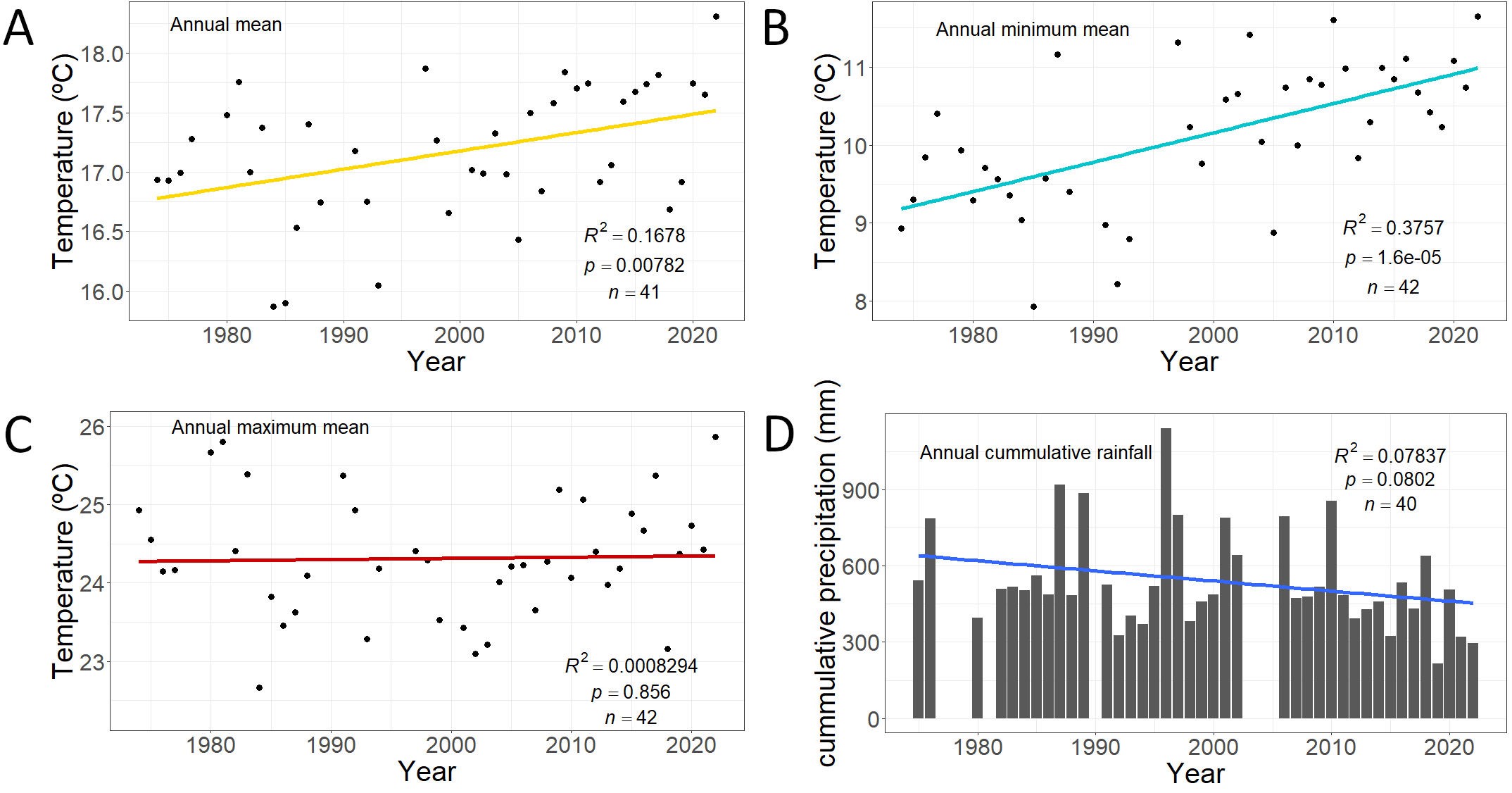

Supplement: mcad193_suppl_Supplementary_Figures_S1 [file mcad193_suppl_supplementary_figures_s1.jpeg]

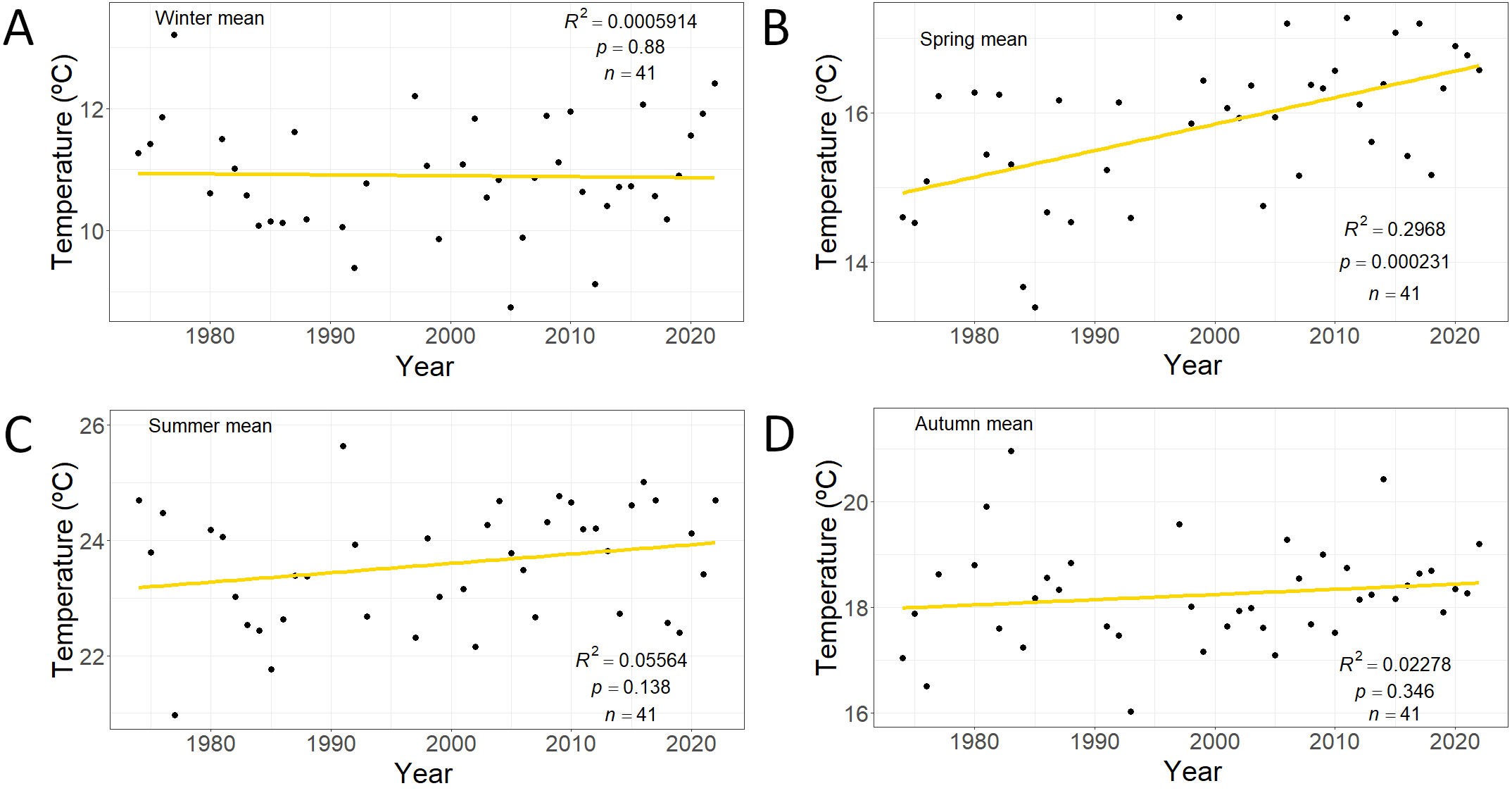

Supplement: mcad193_suppl_Supplementary_Figures_S2 [file mcad193_suppl_supplementary_figures_s2.jpeg]

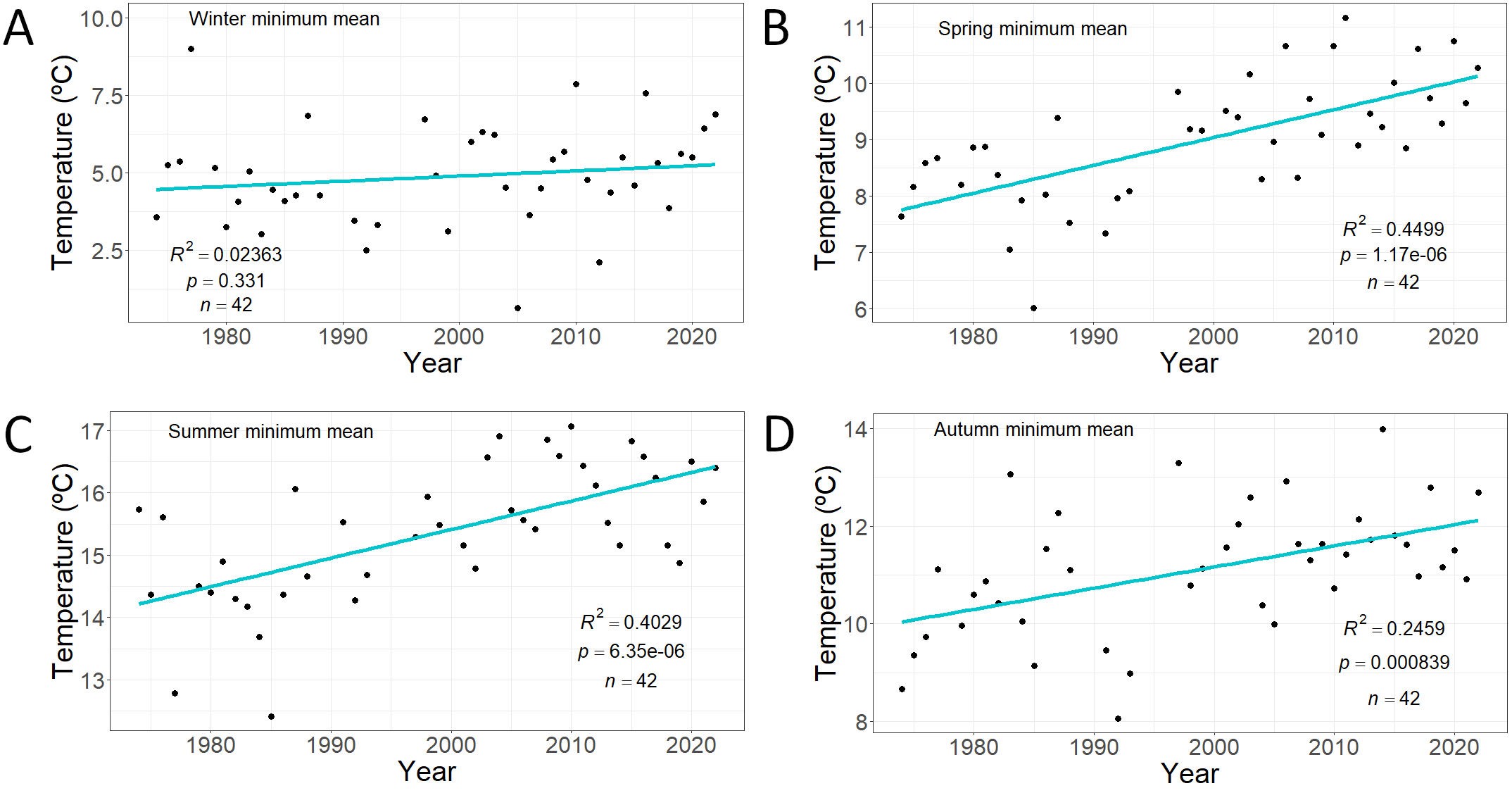

Supplement: mcad193_suppl_Supplementary_Figures_S3 [file mcad193_suppl_supplementary_figures_s3.jpeg]

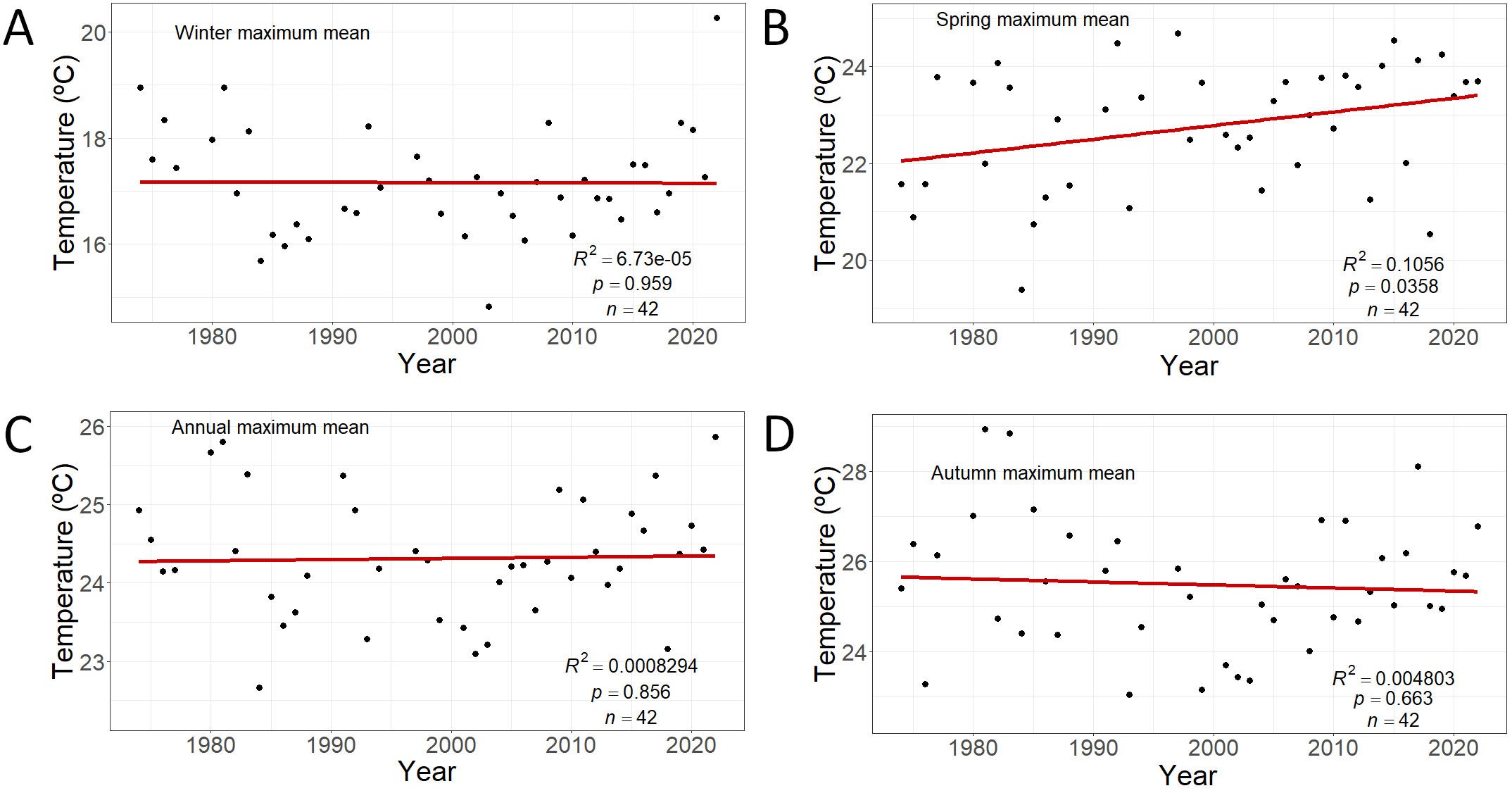

Supplement: mcad193_suppl_Supplementary_Figures_S4 [file mcad193_suppl_supplementary_figures_s4.jpeg]

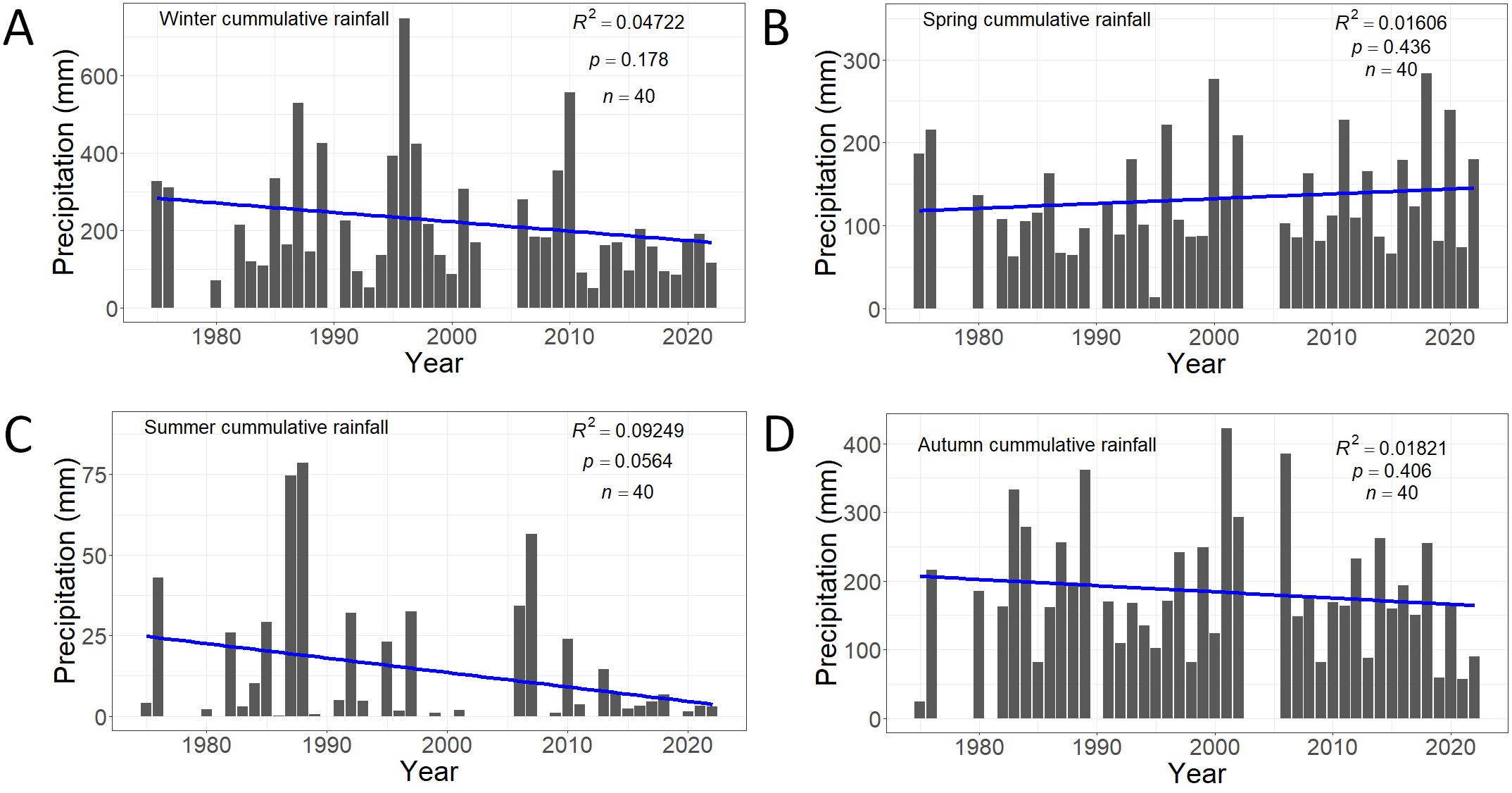

Supplement: mcad193_suppl_Supplementary_Figures_S5 [file mcad193_suppl_supplementary_figures_s5.jpeg]

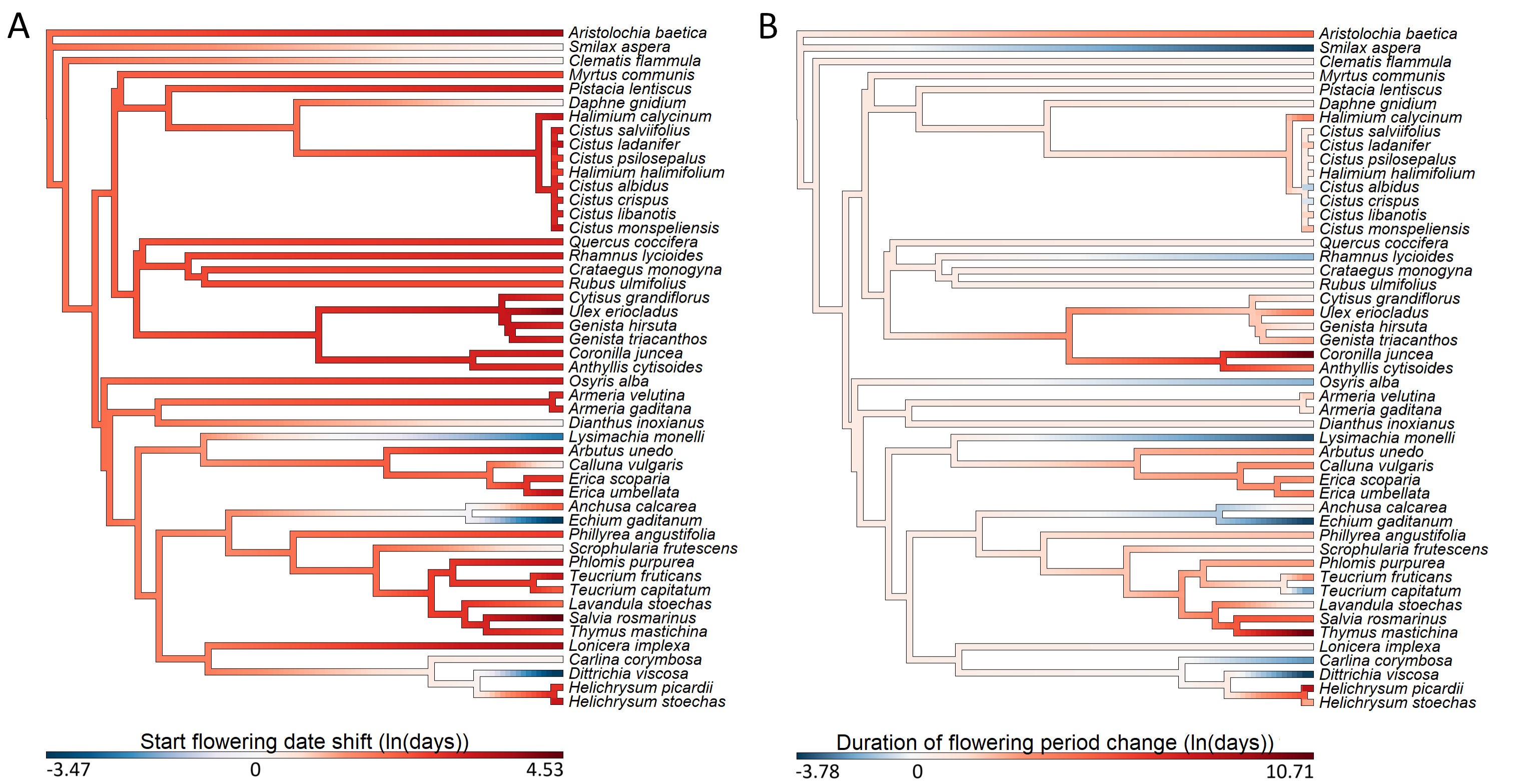

Supplement: mcad193_suppl_Supplementary_Figures_S6 [file mcad193_suppl_supplementary_figures_s6.jpeg]

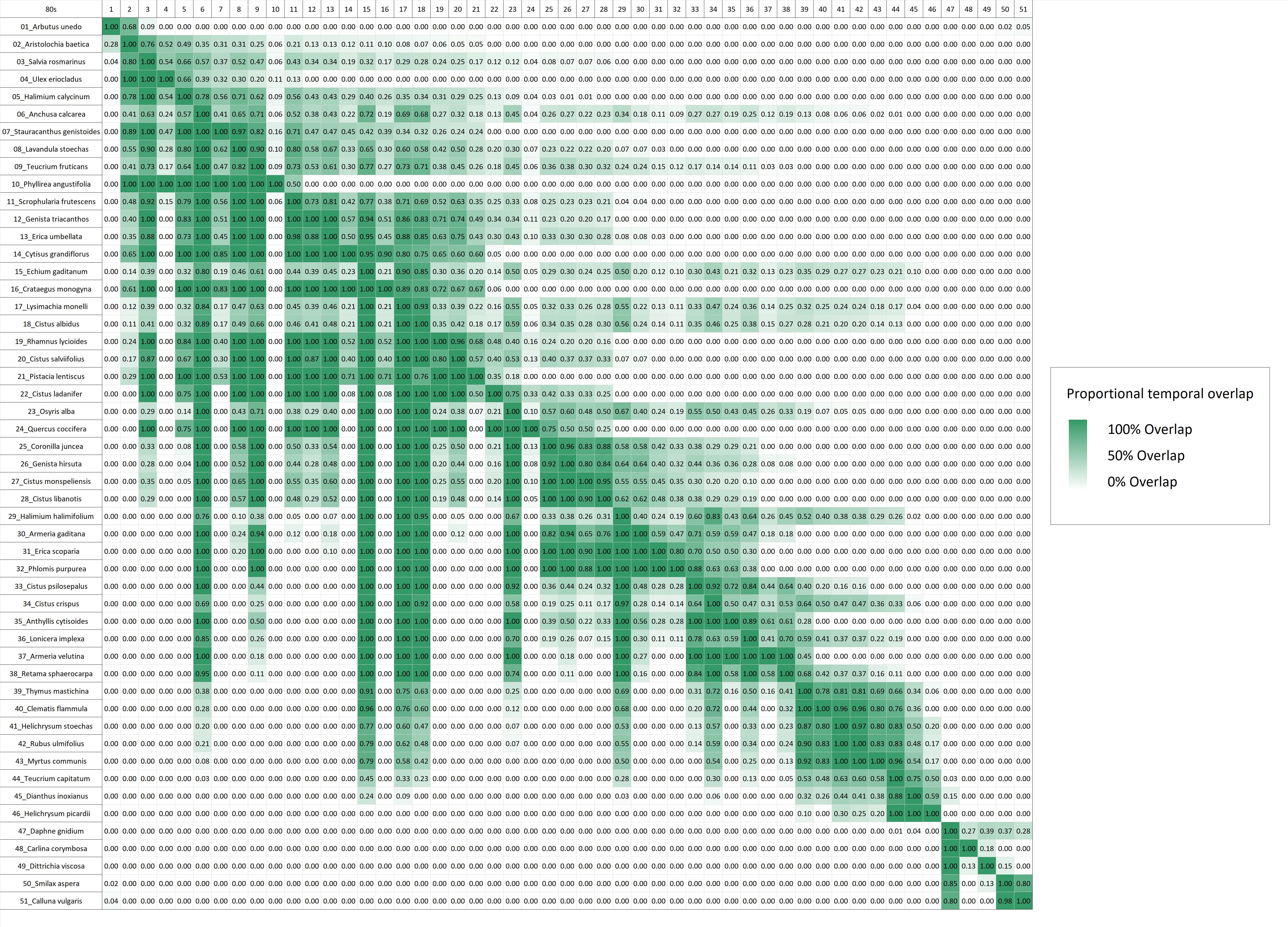

Supplement: mcad193_suppl_Supplementary_Figures_S7 [file mcad193_suppl_supplementary_figures_s7.jpeg]

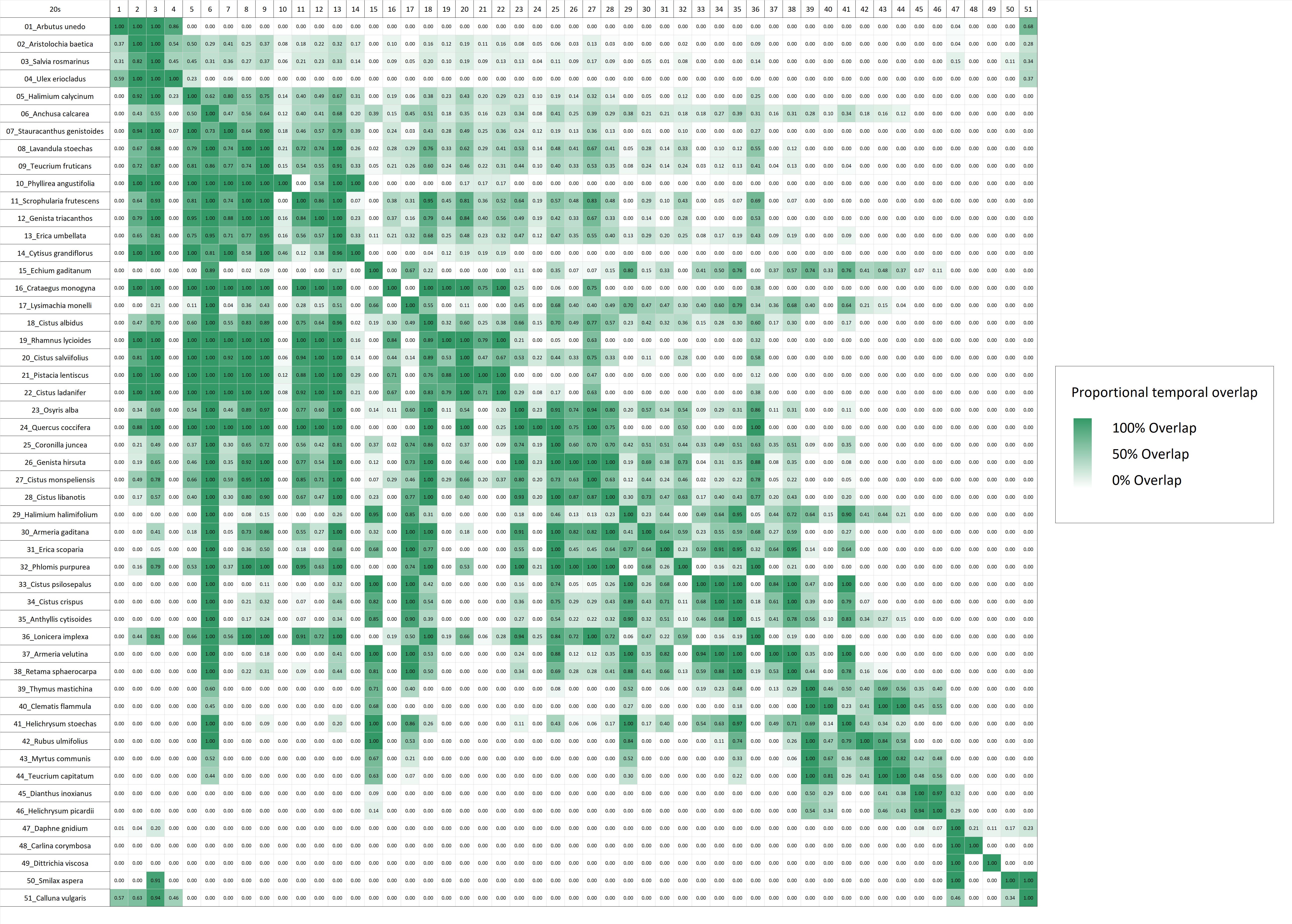

Supplement: mcad193_suppl_Supplementary_Figures_S8 [file mcad193_suppl_supplementary_figures_s8.jpeg]

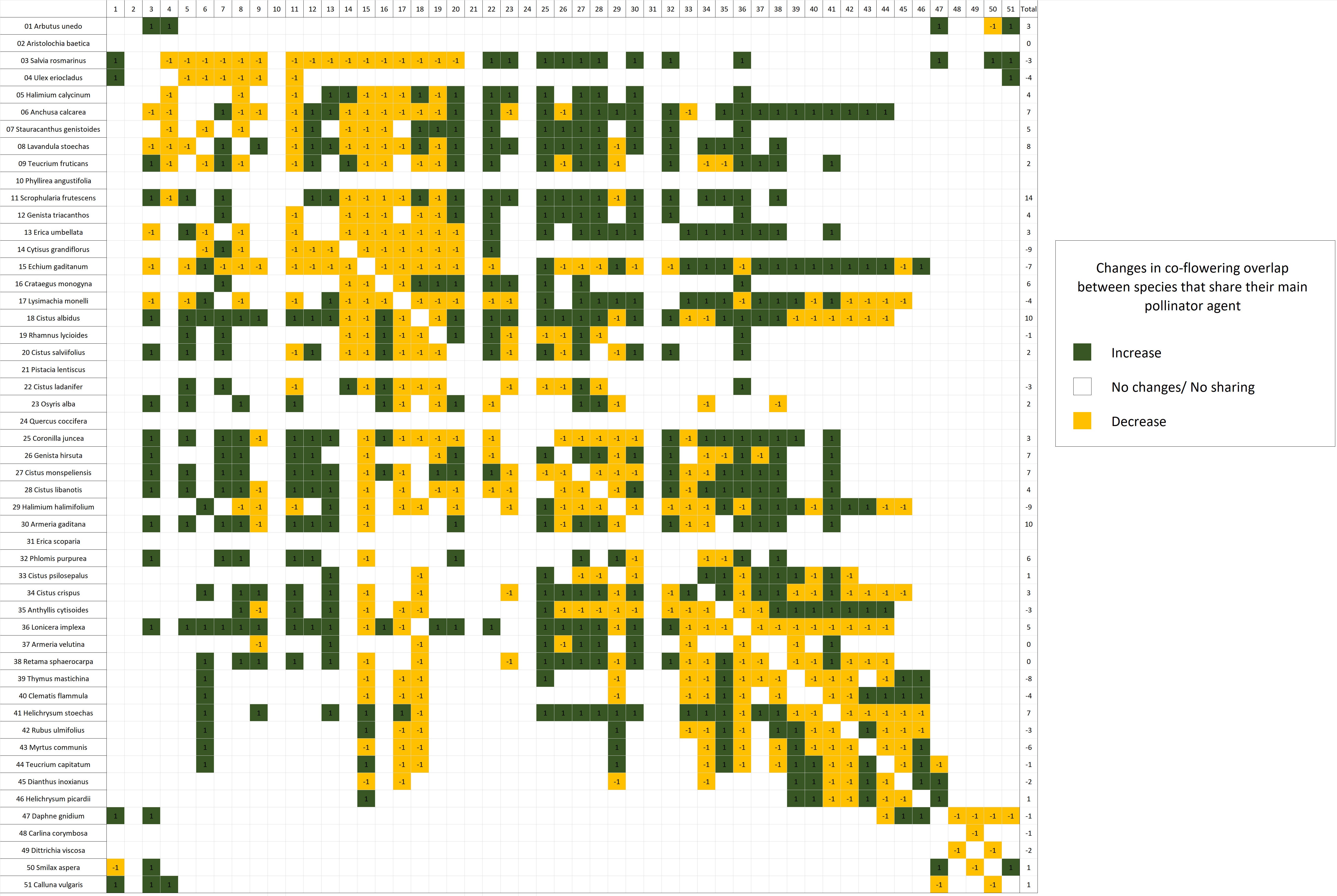

Supplement: mcad193_suppl_Supplementary_Figures_S9 [file mcad193_suppl_supplementary_figures_s9.jpeg]
